# Real-World Outcomes of Autologous Osteoblast Implantation in Patients With Osteonecrosis of the Femoral Head: A Retrospective, Multicenter, Single-Arm Cohort Study in India

**DOI:** 10.7759/cureus.113211

**Published:** 2026-07-23

**Authors:** Gauresh Palekar, Kankanala J Reddy, Aashish Arbat, Prashant Gedam, A. Navaladi Shankar, Gautam Kodikal, Rajkumar Amaravati, Amit Agarwal, Atul Mishra, Alok C Agrawal, Bishnu P Patro, Sridhar Archik, Tejas Gandhi, K. N Subramanian, Rahul Damle, Narayan Hulse, Subodh Mehta, Abhijeet Chandge, Manish Maheshwari, Nandan Rao, Aniket Shah

**Affiliations:** 1 Department of Orthopaedics, Jaslok Hospital and Research Center, Maharashtra, IND; 2 Department of Orthopaedics, Surya Orthopedic Clinic, Girgaon, IND; 3 Department of Orthopaedics and Traumatology, Apollo Hospitals, Hyderabad, IND; 4 Department of Orthopaedics, Jehangir Hospital, Pune, IND; 5 Department of Orthopaedics, Topiwala National Medical College (TNMC) and Bai Yamunabai Laxman (BYL) Nair Charitable Hospital, Mumbai, IND; 6 Department of Orthopaedics, Apollo Hospitals, Chennai, IND; 7 Department of Orthopaedics, Apollo Spectra Hospitals - Koramangala, Bengaluru, IND; 8 Department of Orthopaedics, St. John Hospital, Bengaluru, IND; 9 Department of Orthopaedics, Indraprastha Apollo Hospitals, New Delhi, IND; 10 Department of Orthopaedics, Fortis Hospital, Noida, Noida, IND; 11 Department of Orthopaedics, All India Institute of Medical Sciences, Raipur, Raipur, IND; 12 Department of Orthopaedics, All India Institute of Medical Sciences, Bhubaneswar, Bhubaneswar, IND; 13 Department of Orthopaedics, Lilavati Hospital & Research Centre, Mumbai, IND; 14 Department of Orthopaedics, Arihant Orthopedic Hospital, Ahmedabad, IND; 15 Department of Orthopaedics, Vale Hospital, Madurai, IND; 16 Department of Orthopaedics, Fortis Hospital, Bannerghatta Road, Bengaluru, IND; 17 Department of Orthopaedics, Advanced Orthopedic and Joint Replacement Centre, Thane, IND; 18 Department of Orthopaedics, Dr Hedgewar Rugnalaya, Chhatrapati Sambhajinagar, IND; 19 Department of Orthopaedics, Shalby Multi-Specialty Hospital, Indore, IND; 20 Department of Orthopaedics, Unity Hospital, Surat, IND; 21 Department of Orthopaedics, Sapphire Multispeciality Hospital, Nashik, IND

**Keywords:** avascular necrosis, hip osteonecrosis, hip preservation, orthobiologics, osteoblast cell implantation, osteoblasts, osteonecrosis of femoral head, stem-cells

## Abstract

Background

Recent advancements in orthobiologics suggest that osteoblast cell therapy combined with core decompression is an emerging approach for the treatment of osteonecrosis of the femoral head (ONFH). This study aimed to assess clinical outcomes in patients with ONFH who underwent osteoblast cell therapy with core decompression.

Methods

This retrospective, multicenter, single-arm cohort study included patients who underwent osteoblast cell therapy for the treatment of stages I, II, and IIIA ONFH between April 2017 and April 2022, with a follow-up of two to seven years. The primary outcomes included improvements in pain and functional capacity, measured using the Visual Analogue Scale (VAS) score and Harris Hip Score (HHS). The secondary outcome was treatment failure, defined as patients undergoing surgical treatment, including but not limited to total hip arthroplasty (THA), as assessed by the surgeon.

Results

The study included 319 patients (mean age: 34.4 ± 9.2 years; male: n = 272, 85.3%). Of these, 183 (57.4%) had bilateral ONFH. Of 502 treated hips, most had stage II (n = 226; 45.0%) or stage IIIA (n = 215; 42.8%) ONFH. The mean follow-up was 3.5 years (42.0 ± 5.2 months). The mean change in VAS score (3.78; 95% CI: 3.57-3.99) and HHS (47.01; 95% CI: 45.13-48.89) at follow-up was significant and exceeded the presupposed minimal clinically important difference (MCID). At follow-up, 31 patients (44 hips) required THA; no patient required any other surgical treatment. The patient-wise and hip-wise failure rates were 9.7% and 8.8%, respectively, with a median THA-free survival of 78 months.

Conclusions

Core decompression with adjuvant osteoblast cell therapy was associated with reduced pain and improved functional capacity in patients with stage I-IIIA ONFH while maintaining femoral head integrity.

## Introduction

Osteonecrosis of the femoral head (ONFH) is a progressive, degenerative disease of the hip characterized by microfractures of the subchondral bone and collapse of the femoral head, which ultimately leads to hip dysfunction [1]. It predominantly affects young or middle-aged individuals. Epidemiological studies have demonstrated that steroid intake, trauma, and chronic alcohol intake are common etiologies of ONFH; however, in nearly 20-30% of cases, the cause of ONFH is idiopathic [2,3].

At the cellular level, osteoblasts and osteocytes play a critical role in osteogenesis. However, in ONFH, osteocyte apoptosis, lipid accumulation in osteoblasts and osteocytes, and decreased osteoblastic differentiation potential of mesenchymal stem cells (MSCs) near the ONFH lesion led to inadequate bone repair [4-8]. Additionally, prolonged ischemia during the course of the disease further reduces osteoblast concentration. Together, these processes lead to further disease progression, loss of structural integrity, and subchondral fracture of the femoral head [9].

Treatment for ONFH aims to relieve pain, retard disease progression, prevent joint collapse, and restore hip biomechanics. In the early stages of ONFH, non-surgical treatment approaches such as restricted weight bearing, pharmacological therapy, and non-invasive biophysical modalities (electromagnetic stimulation, extracorporeal shock-wave therapy, and hyperbaric oxygen) are often recommended [2,10]. However, in patients with advanced disease, surgical interventions become necessary. Repair and reconstruction methods such as core decompression, osteotomy, and non-vascularized bone transplantation aim to preserve the femoral head. If these methods are ineffective, total hip arthroplasty (THA) may be considered [11].

Core decompression is a widely used and safe surgical treatment for early-stage ONFH. This procedure decreases intra-osseous pressure, improves vascularity to the necrotic area, and augments new bone formation before mechanical failure of the femoral head [12,13]. However, core decompression alone may not provide structural support, likely due to a lack of osteoprogenitor cells in the femoral head. This limitation increases the risk of femoral head collapse and leads to clinical failure in about 30% of patients with ONFH [14,15]. The implantation of orthobiologics such as platelet-rich plasma, bone marrow aspirate concentrate, stromal vascular fraction, and autologous cultured osteoblast cells, along with core decompression, is an innovative strategy for the treatment of ONFH [10,14,16]. Osteoblast cell therapy involves ex vivo processing of the autologous bone marrow to isolate and differentiate MSCs into osteoblast cells and further expand them before implantation [16]. Various clinical studies have demonstrated delayed progression to subchondral fracture and reduced pain in patients with ONFH treated with combined core decompression and osteoblast cell therapy [16,17].

Although previous studies have demonstrated the safety and efficacy of this approach for the treatment of ONFH, these had small patient populations and short follow-up durations [16,17]. Hence, the present study was conducted to assess clinical outcomes (pain intensity, functional capacity, and the need for other surgical treatments) in patients who underwent osteoblast cell therapy with core decompression for the treatment of ONFH at different clinical centers across India. The study was carried out following the market authorization of OSSGROW® (Regrow Biosciences Pvt. Ltd., Mumbai, Maharashtra), which comprises autologous adult live-cultured osteoblasts (AALCO), referred to as osteoblast cell therapy, by the Drugs Controller General of India, Ministry of Health and Family Welfare, Government of India, in 2017 [18].

## Materials and methods

This was a retrospective, multicenter, single-arm cohort study that included data from patients who underwent osteoblast cell therapy for ONFH between April 2017 and April 2022 at 21 tertiary care centers across India. The participating centers were selected based on the investigators' expertise in the management of ONFH and hip reconstruction procedures, geographic spread across India, inclusion of both public and private healthcare centers, and willingness to contribute retrospective patient data for the study. The study was approved by the Regrow Biosciences Independent Ethics Committee (letter number: REG/IEC/2025/006), and was conducted in accordance with Good Clinical Practice (GCP) and the Declaration of Helsinki. 

Patient population

This study included patients aged 18-52 years, diagnosed with stages I, II, and IIIA of ONFH of multifactorial etiology, according to the 2019 revised version of the Association Research Circulation Osseous (ARCO 2019) classification [19]. Additionally, patients included were those who had previously been treated with osteoblast cell therapy and were followed up for two to seven years. Patients who had undergone any treatment for cancer over the last two years or had an active infection were excluded. Patients with neurological or psychiatric conditions, cardiac abnormalities, genetic disorders, or hemoglobinopathies were also excluded.

No predefined sample size calculation was performed. Instead, all patients who met the inclusion criteria within the defined study period were included. 

Study procedure

The diagnosis of ONFH and disease stage were confirmed based on findings of X-ray and magnetic resonance imaging (MRI). After osteoblast cell therapy, patients were followed up clinically at three, six, and 12 months, and then annually to assess pain, functional outcomes, and the need for additional surgeries. Retrospective data of the patients were extracted from progress notes, discharge summaries, and radiology reports. The coronavirus disease 2019 (COVID-19)-associated ONFH subgroup was identified retrospectively from hospital medical records. Patients with a history of steroid exposure were excluded from this subgroup to avoid overlapping with steroid-associated ONFH. The interval between COVID-19 infection/recovery and ONFH diagnosis was calculated from the recorded dates. During the COVID-19 pandemic (early 2020 to mid-2021), patients were followed up telephonically.

Surgical procedure and rehabilitation

Osteoblast cell therapy involves a two-stage surgical procedure performed under spinal anesthesia. The details of the surgical procedure and rehabilitation have been described previously [16]. In the first stage, 4-8 mL of bone marrow was aspirated and collected from the posterior superior iliac crest and transported to a Good Manufacturing Practices (GMP)-certified facility (Regrow Biosciences Pvt. Ltd., Lonavala, Pune) for ex vivo expansion.

Thereafter, MSCs from the bone marrow were centrifuged, resuspended in a growth medium containing L-ascorbic acid and other growth factors, allowed to differentiate into osteoblastic lineage cells, and expanded over three to four weeks. Cells were stained with anti-human bone alkaline phosphatase antibodies (BioLegend, San Diego, California, United States) and characterized using flow cytometry (Attune NXT; Thermo Fisher Scientific Inc., Waltham, Massachusetts, United States). Additionally, cells were subjected to Alizarin Red staining to confirm osteoblastic phenotype. After passing release testing criteria (negative for endotoxin, bacterial, mycoplasma, and other viable impurities), a minimum of 48 million viable osteoblast cells were transported back for implantation, maintaining the cold chain.

**Figure 1:**
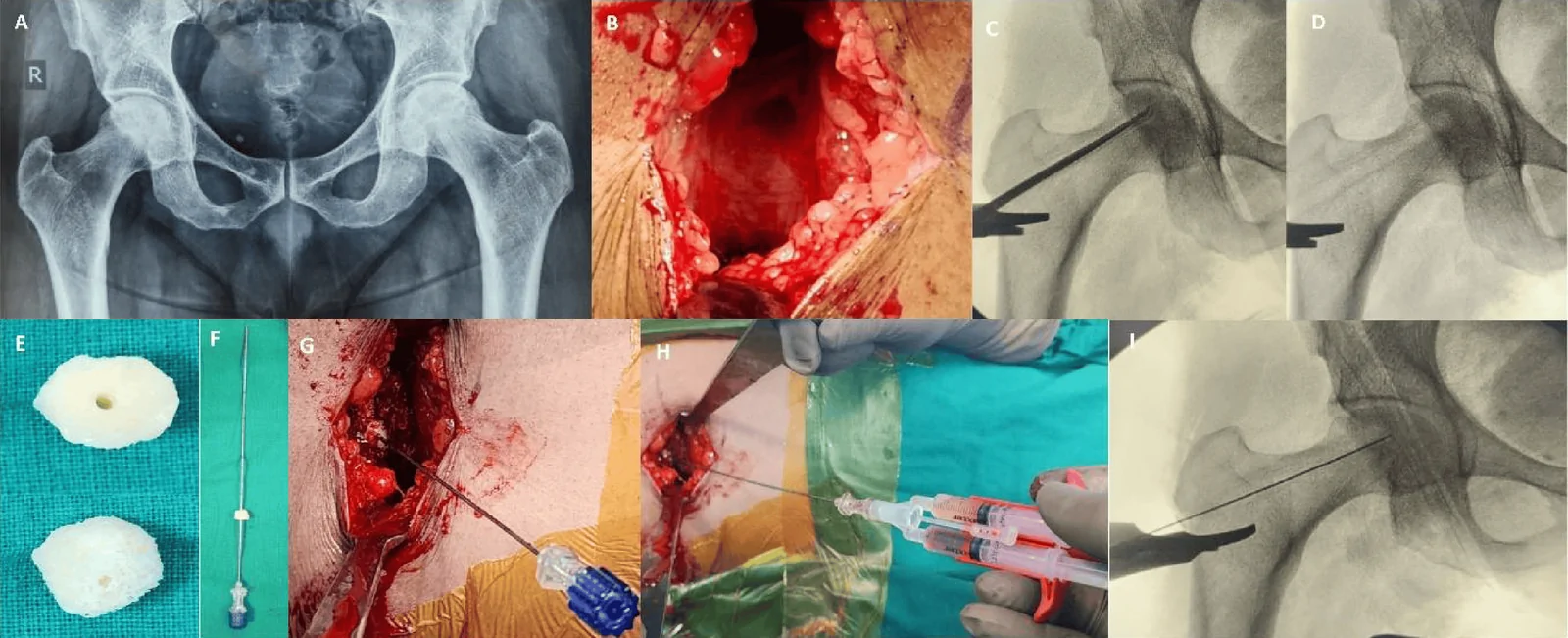
Pre- and intraoperative images of osteoblast cell therapy (A) Preoperative X-ray of a 47-year-old male patient with ONFH; (B) Incision and opening for passing the guide wire; (C, D) Core decompression, a standard procedure, was performed under C-arm guidance, and angular curettes were used to remove sclerotic necrotic bone, creating space for new bone formation; (E) Allograft plug; (F) Allograft plug placed onto the long spinal needle (CE-marked; 18G/20 cm); (G) Long spinal needle positioned for final product implantation; (H, I) Final osteoblast cell–gel mixture delivered to the necrotic site for bone regeneration CE: Conformité Européenne (indicating compliance with European requirements that ensure safety, health, and environmental protection standards); ONFH: osteonecrosis of the femoral head

In the final operative step, patients were placed supine on a fracture table, with the affected limb internally rotated by 15 degrees. The lesion was marked in both anteroposterior and lateral planes using 2 mm K-wires (Kirschner wires) under C-arm guidance. Core decompression was performed using an 8 mm cannulated drill over the guide wires, and the sclerotic bone at the lesion site was thoroughly removed using curettage. A total of 4.8 × 10^7^ autologous live cultured osteoblasts were delivered into the decompressed area using a certified fibrin glue (TISSEEL; Baxter International Inc., Deerfield, Illinois, United States) and an 18-gauge spinal needle via an eight mm interference screw. Finally, the tract was sealed with cancellous allograft obtained from a certified bone bank (Figure 1). A standardized surgical procedure manual was provided to all participating centers in the study.

After implantation, patients were instructed to perform partial weight-bearing exercise for four weeks using a walker. Patients were allowed to walk using a stick by Week 6 and then to bear full weight by Week 8. However, in the case of bilateral ONFH, a walker was needed until Week 6. Passive lower-limb exercises to regain muscle strength and hip joint movements were performed as early as possible.

Study outcomes

The primary outcomes included improvement in pain intensity and functional capacity at follow-up from baseline, and the secondary outcome was treatment failure.

Improvements in pain intensity at the patient level were assessed using the patient-reported Visual Analogue Scale (VAS) (range, 0-10, with 10 indicating the worst imaginable pain) [20]. Functional capacity was assessed by the treating physician at the patient level using the Harris Hip Score (HHS) (0-100 points, with > 90: excellent, 80-90: good, 70-80: okay, and < 70: poor outcomes) [21]. The minimal clinically important difference (MCID) thresholds were predefined as two points for VAS and 10 points for HHS. Treatment failure was defined as the patients undergoing surgical treatment, including but not limited to THA, as assessed by the surgeon. The follow-up time for hip-level analyses, such as THA conversion and time-to-THA, was defined as the interval from osteoblast cell therapy to THA conversion or censoring at the last known follow-up; bilateral hips were treated as separate observations with patient-level clustering. Patients were diagnosed using an X-ray or MRI, which revealed a fracture, arthritis, collapse beyond 2 mm, or progression to grade IIIb/IV; these patients generally progressed to THA surgery.

Statistical analysis

Data were summarized using descriptive statistics. Continuous data were represented as mean ± standard deviation (SD), median (interquartile range (IQR)), and were compared using a Student’s t-test or a non‑parametric test, as applicable. Categorical data were presented as frequencies and percentages, and the chi-square test was used for comparative analysis. A p-value of < 0.05 was considered statistically significant. To account for within-patient correlation, additional analyses using cluster-robust standard errors and shared-frailty models were performed. The normality of VAS and HHS scores was assessed using the Shapiro-Wilk and Kolmogorov-Smirnov tests. Failure-free survival for THA was evaluated using Kaplan-Meier (KM) curves, and median follow-up duration was estimated using the reverse KM method. The log-rank test was used to compare different groups based on etiology and stage. The primary analysis for hip-level outcomes was performed using an adjusted Cox proportional hazards model to evaluate THA conversion with robust standard errors clustered by patients. Results were presented as hazard ratios (HRs) and 95% confidence intervals (CIs). Proportional hazards assumptions were evaluated using Schoenfeld residuals. The Cox model adjusted for age, sex, stage, etiology, bilaterality, baseline HHS and VAS, and center effect (modeled via robust variance/frailty), where ARCO stage was modeled as a three-level categorical variable (Stage I reference). Etiology was modeled as a multi-level categorical variable, with alcohol used as the reference category. As a secondary analysis, THA conversion was evaluated using multiple logistic regression with a generalized linear model. All analyses were performed using Python v3.12.12 (Python Software Foundation, Wilmington, Delaware, United States) in Google Colab (Google LLC, Mountain View, California, United States).

## Results

Baseline characteristics

A total of 347 patients (544 hips) treated with combined osteoblast cell therapy and core decompression for ONFH were initially included in the study. However, 28 patients (42 hips) were lost to follow-up; therefore, the final analysis included 319 patients (502 hips). Baseline characteristics were comparable between included patients and those lost to follow-up (all standardized mean differences ≤ 0.44).

The mean age of patients was 34.4 ± 9.2 years, and the majority were male (n = 272; 85.3%). Of the treated patients, 183 (57.4%) had bilateral ONFH (Table 1). Moreover, 214 (42.6%) hips had steroid-induced ONFH, and 188 (37.5%) had idiopathic ONFH. Most of the treated hips were classified as either stage II (n = 226; 45.0%) or stage IIIA (n = 215; 42.8%), with fewer hips in stage I (n = 61; 12.2%) according to the ARCO 2019 classification (Table 2). Baseline characteristics of patients who were lost to follow-up were similar to those of patients included in the study (data not shown). After osteoblast cell therapy, patients were followed up for two to seven years, with an average follow-up duration of 3.5 years (42.0 ± 5.2 months).

**Table 1:** Demographics of the study population ^a^Number of hips = 502 BMI: body mass index; ONFH: osteonecrosis of the femoral head

Parameters	Number of patients (n = 319)^a^
Age (years), mean ± SD	34.4 ± 9.2
BMI (kg/m^2^), mean ± SD	25.7 ± 4.4
Duration since diagnosis (years), mean ± SD	0.7 ± 0.7
Sex, n (%)	
Male	272 (85.3)
Female	47 (14.7)
Unilateral/bilateral ONFH, n (%)	
Unilateral	136 (42.6)
Bilateral	183 (57.4)

**Table 2:** Baseline clinical characteristics of the study population Number of hips = 502; ^a^Calculations were based on the number of treated hips = 502; ^b^Stages I and II are precollapse stages, whereas stage IIIA is an early post-collapse (less than two mm collapse) stage. ARCO 2019: 2019 revised version of the Association Research Circulation Osseous; COVID-19: coronavirus disease 2019; ONFH: osteonecrosis of the femoral head; IQR: interquartile range

Parameters	Frequency (Percentage)	Median (IQR) (months)
Stages of the hip per ARCO 2019^a,b^		
Stage I	61 (12.2)	42.0 (38.0-47.0)
Stage II	226 (45.0)	42.0 (38.0-46.0)
Stage IIIA	215 (42.8)	42.0 (37.0-45.0)
Etiology of ONFH^a^		
Steroid	214 (42.6)	41.0 (38.0-46.0)
Idiopathic	188 (37.5)	43.0 (39.0-46.0)
COVID-19 (without steroids)	52 (10.4)	42.0 (35.2-46.2)
Alcohol	33 (6.6)	40.0 (24.0-47.0)
Trauma	15 (3.0)	43.0 (39.0-49.5)

Clinical outcomes

**Figure 2:**
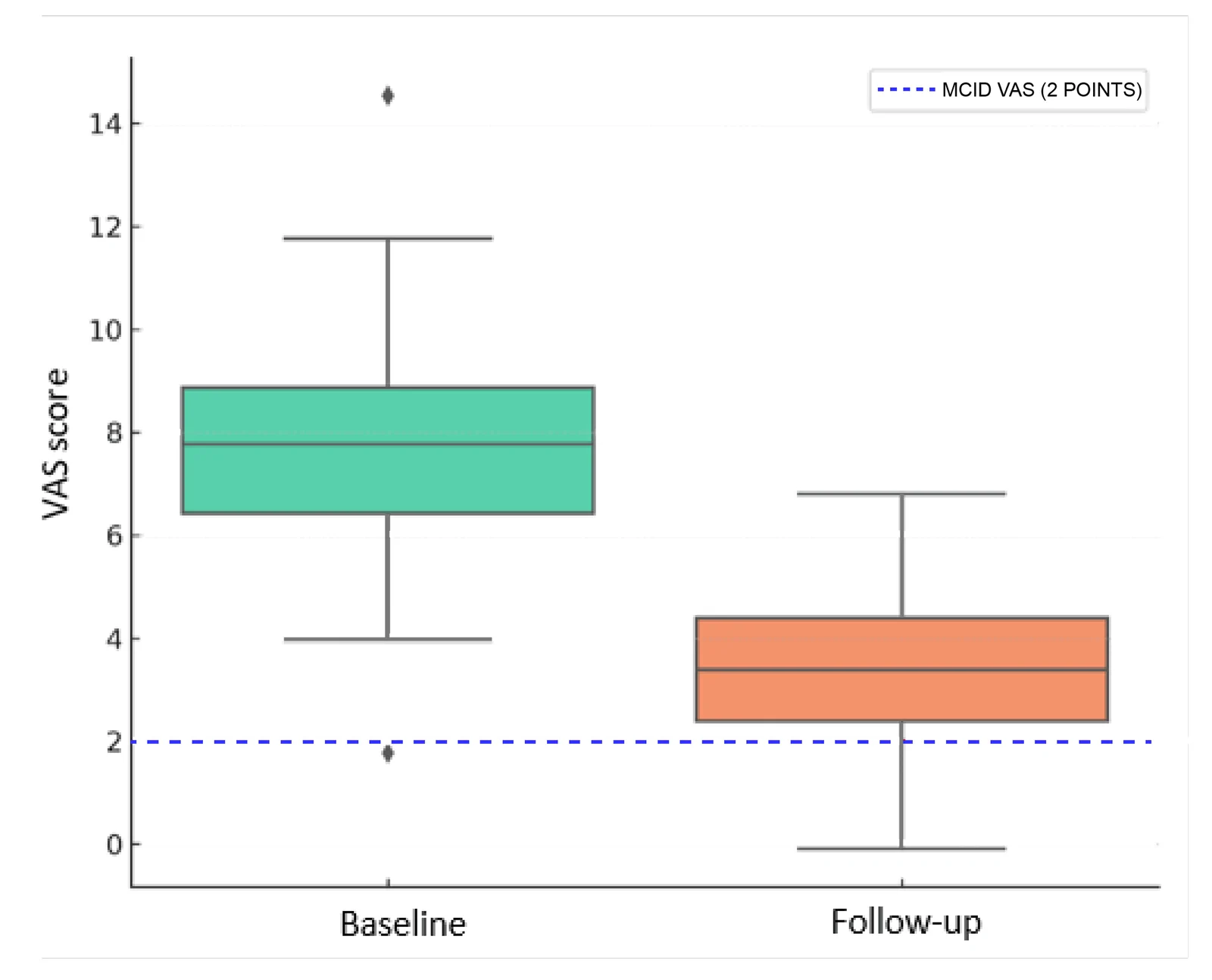
Visual analogue scale score at baseline and follow-up visits Data are presented patient-wise. The blue dotted line indicates the MCID threshold value for VAS. MCID: minimal clinically important difference; VAS: visual analogue scale

At follow-up, 80.6% 80.6% (257/319; 95% CI: 75.90-84.50) of patients showed an improvement of ≥ 2 points in the VAS score, with a mean change of 3.78 (95% CI: 3.57-3.99) and a standard response mean of 2.00 (Figure 2).

**Figure 3:**
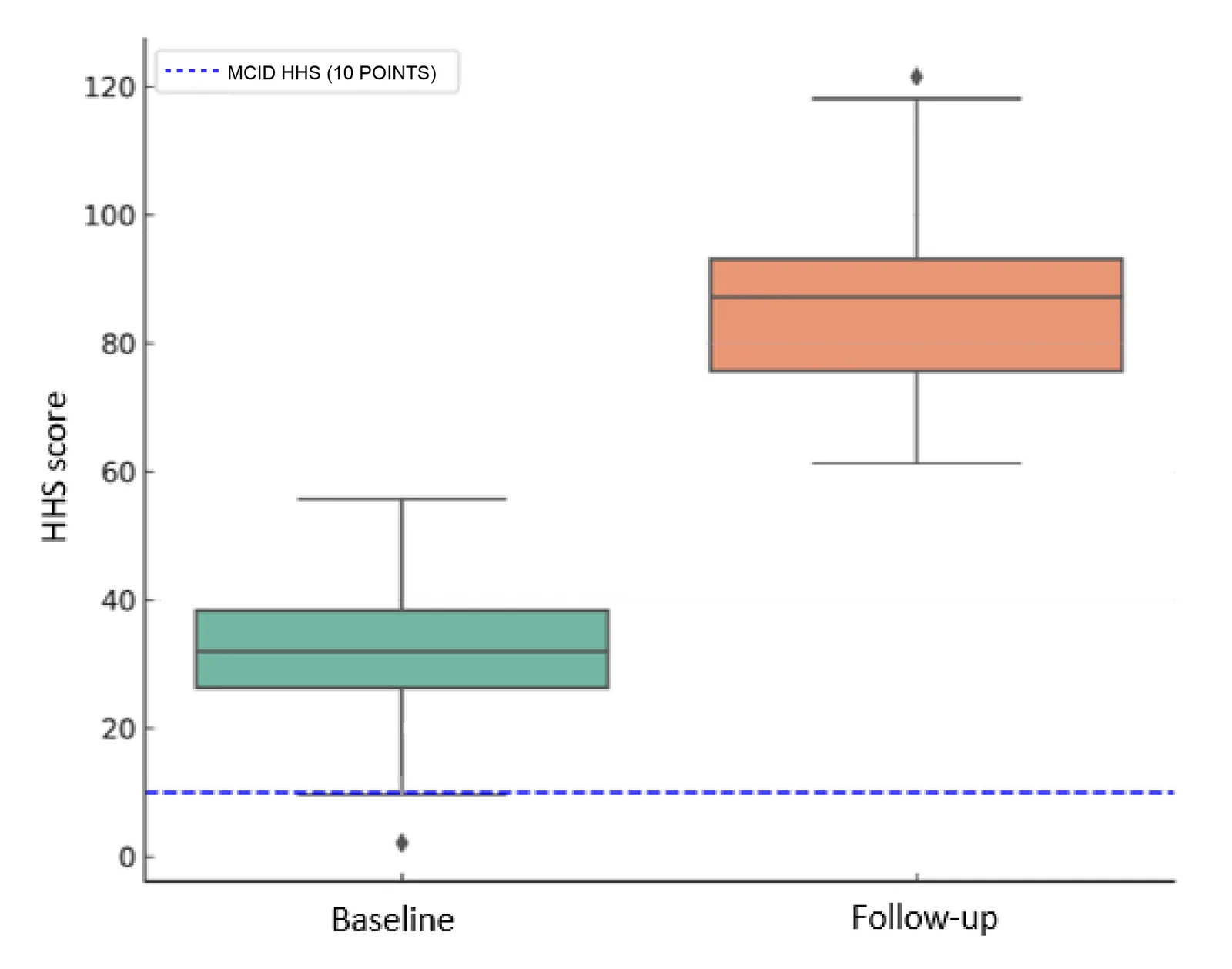
HHS at baseline and follow-up visits Data are presented patient-wise. The blue dotted line indicates the MCID threshold value for HHS. HHS: Harris Hip Score; MCID: minimal clinically important difference

Likewise, 90.3% (288/319; 95% CI: 86.50-93.10) of patients showed an improvement of ≥ 10 points in the HHS, with a mean change of 47.01 (95% CI: 45.13-48.89) and a standard response mean of 2.75 (Figure 3).

**Figure 4:**
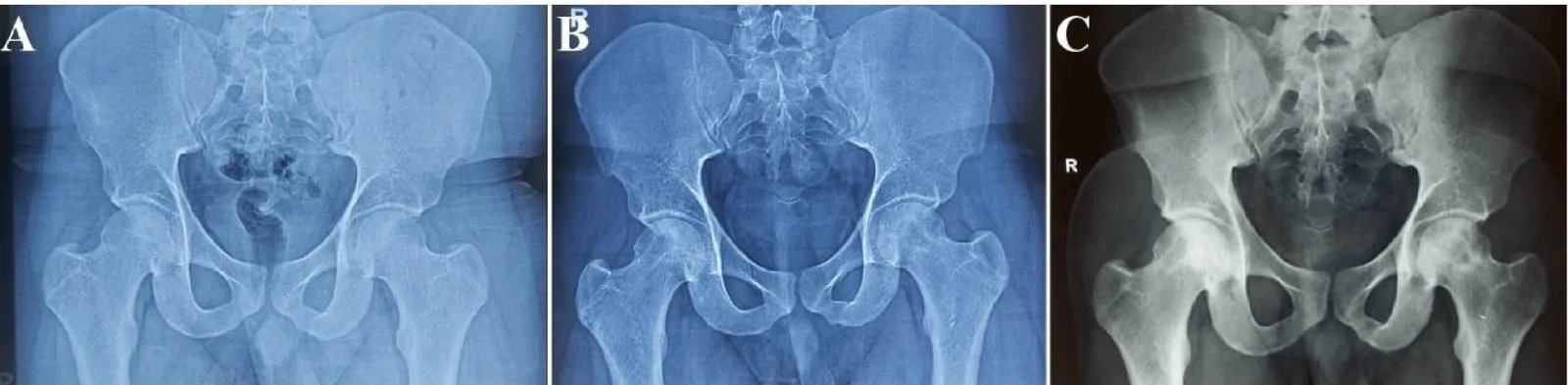
Preoperative and postoperative (9 and 15 months) X-ray of pelvis (A) Preoperative X-ray of the pelvis showing ONFH and normal joint space; (B, C) 9- and 15-month postoperative X-rays with osteoblast cell therapy showing a smooth femoral head surface with normal joint space and minimal sclerosis of the femoral head, suggestive of osteogenesis. ONFH: osteonecrosis of the femoral head

**Figure 5:**
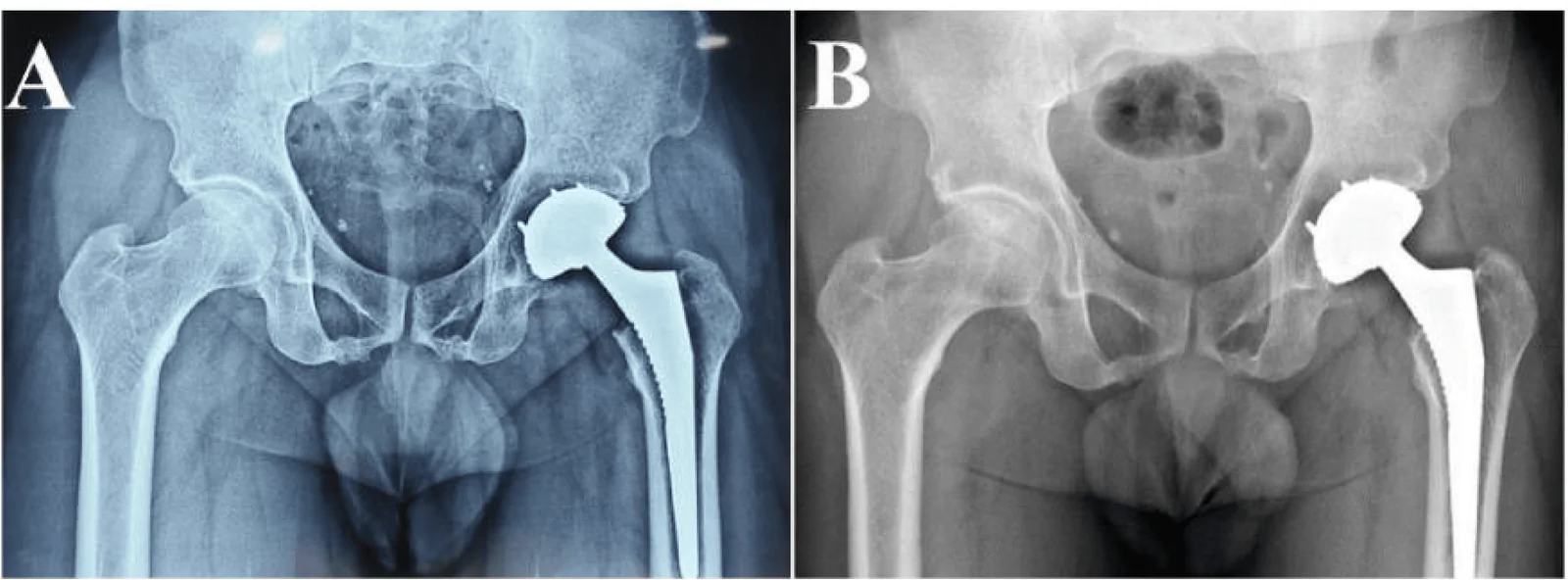
Preoperative and postoperative (24 months) X-ray of pelvis with hips (A) Preoperative X-ray of the pelvis showing the right hip with ONFH and the left hip with a total hip replacement prosthesis in situ; (B) 24-month follow-up X-ray showing sclerosis of the right femoral head indicative of osteogenesis, where core decompression with osteoblast cell therapy was done. ONFH: osteonecrosis of the femoral head

Pre- and postoperative representative X-ray images of patients who underwent osteoblast cell therapy are shown in Figures 4-5. The postoperative images show radiographic improvement after successful osteoblast cell therapy.

**Figure 6:**
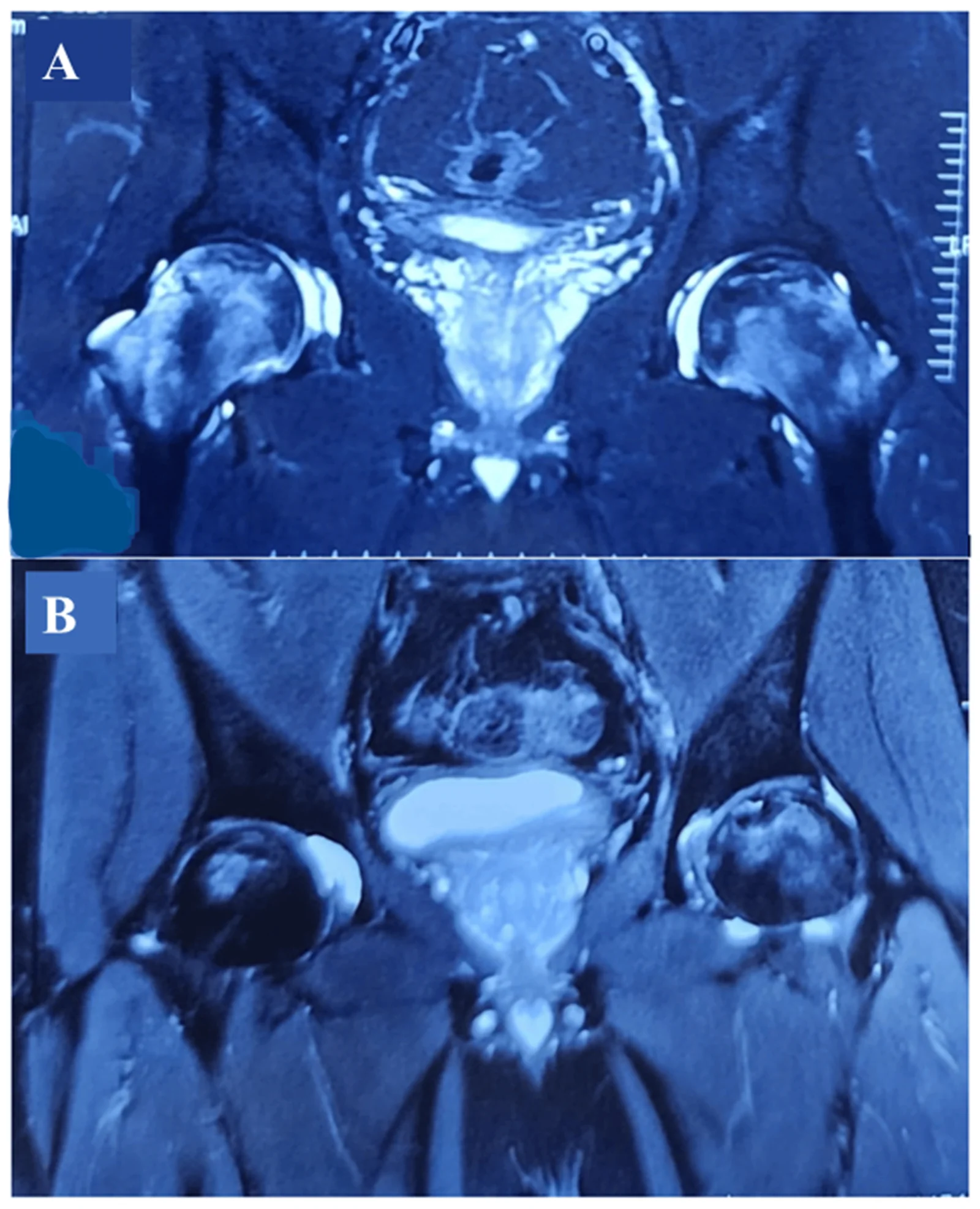
Preoperative and postoperative (24 months) MRI (A) Preoperative MRI; (B) 24-month follow-up MRI showing evidence of osteogenesis in the femoral head, with maintenance of sphericity of the femoral heads and of the joint space. MRI: magnetic resonance imaging

**Figure 7:**
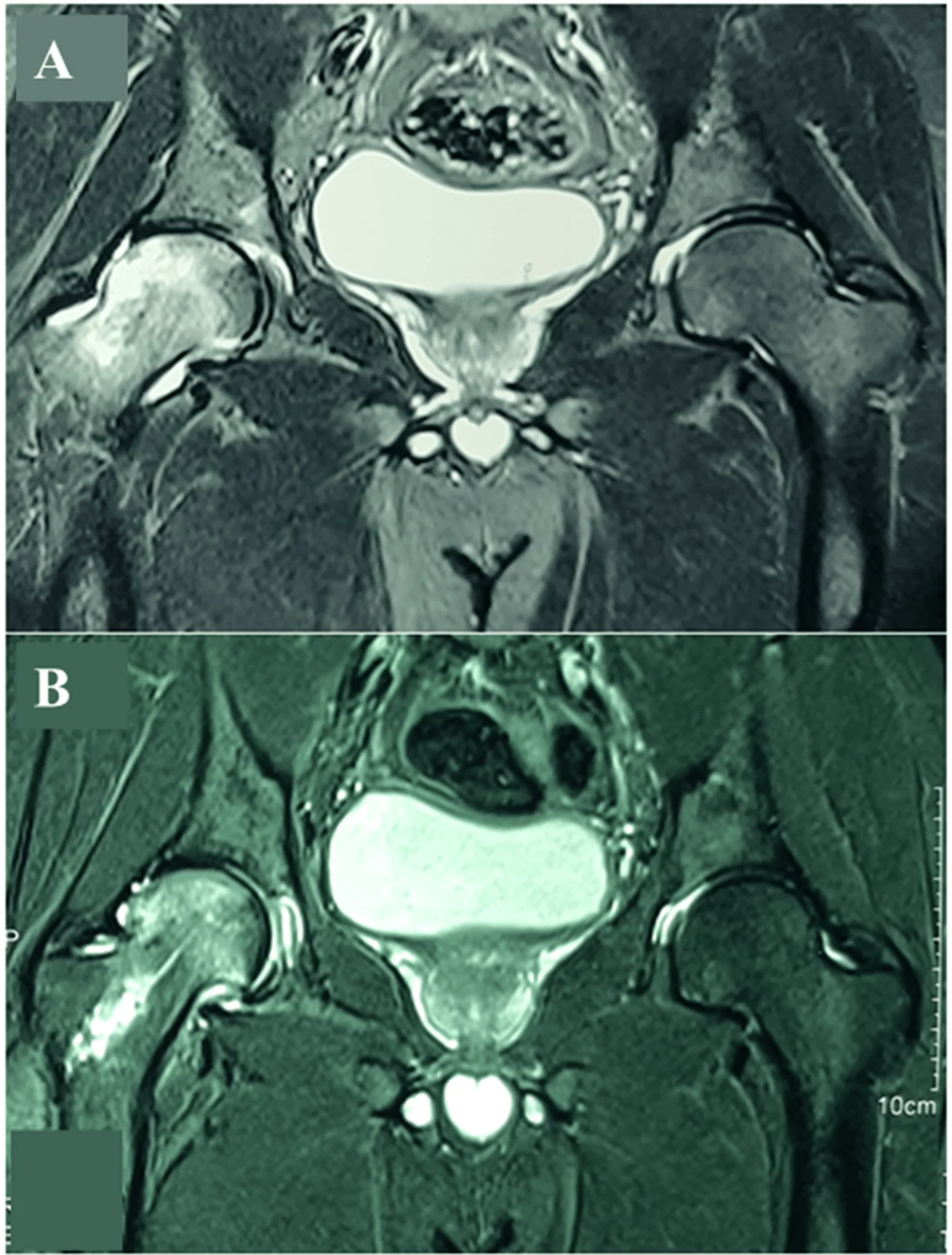
Preoperative and postoperative (36 months) MRI of hips (A) Preoperative MRI of the right hip; (B) 36-month follow-up MRI showing evidence of osteogenesis in the right femoral head, with maintenance of sphericity of right femoral head and of joint space. MRI: magnetic resonance imaging

Likewise, representative postoperative MRI images demonstrated maintenance of femoral heads’ sphericity and joint spaces, with no further collapse; there were no clear radiological signs of progression of disease or arthritic changes in the femoral head (Figures 6-7).

**Figure 8:**
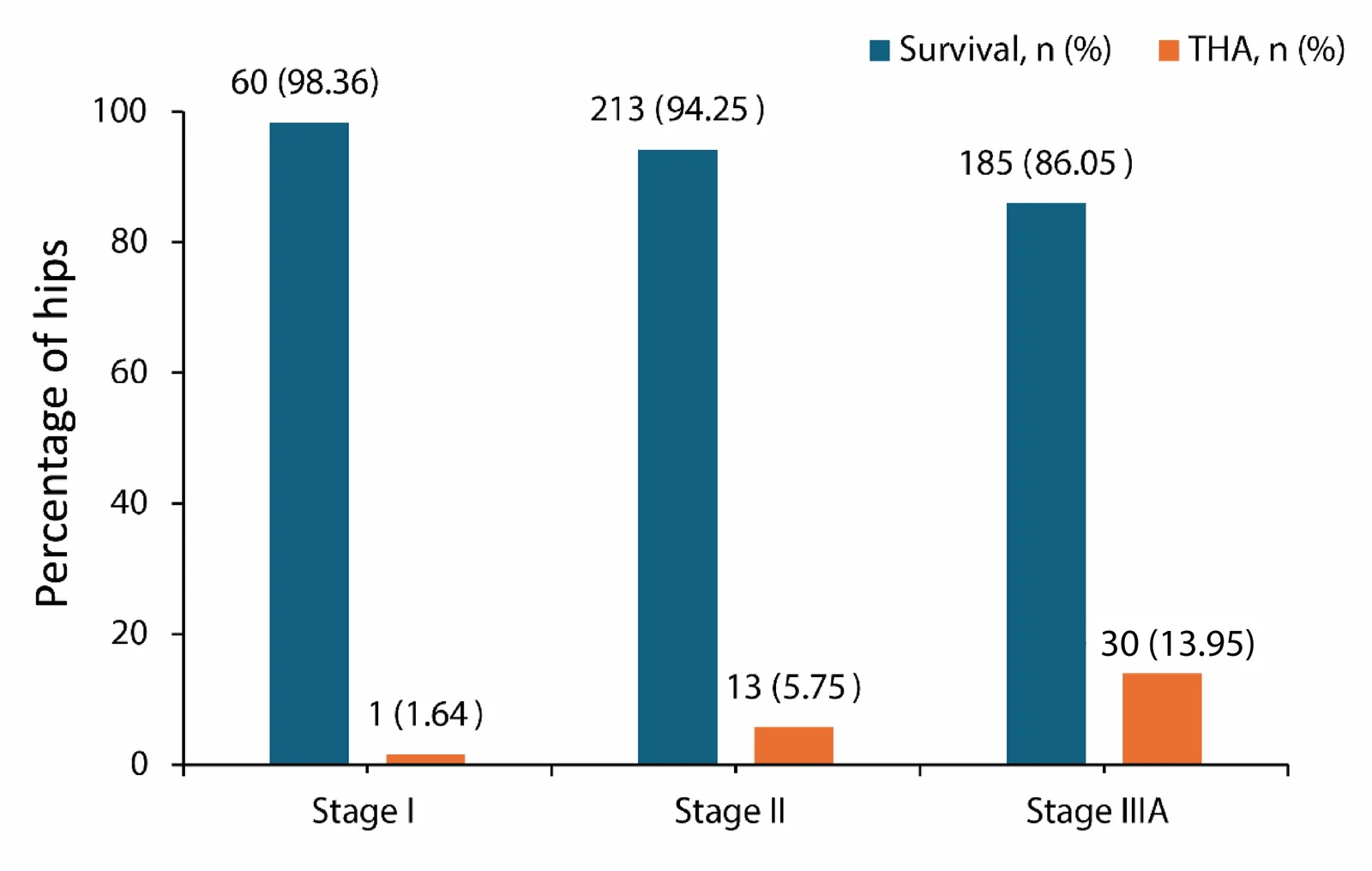
Stage-wise hip survival vs failure after osteoblast cell therapy THA: total hip arthroplasty.

At follow-up, 31 patients (44 hips) required THA for the hips treated with osteoblast cell therapy. No patient required any other surgical treatment for ONFH. Hence, the estimated patient-wise and hip-wise treatment failure rates were 9.7% and 8.8%, respectively. The average time for hip failure was 2.7 years (range: 1-7 years). Of the treated hips, the majority that required THA at follow-up had stage IIIA ONFH (30/44) (Figure 8).

**Figure 9:**
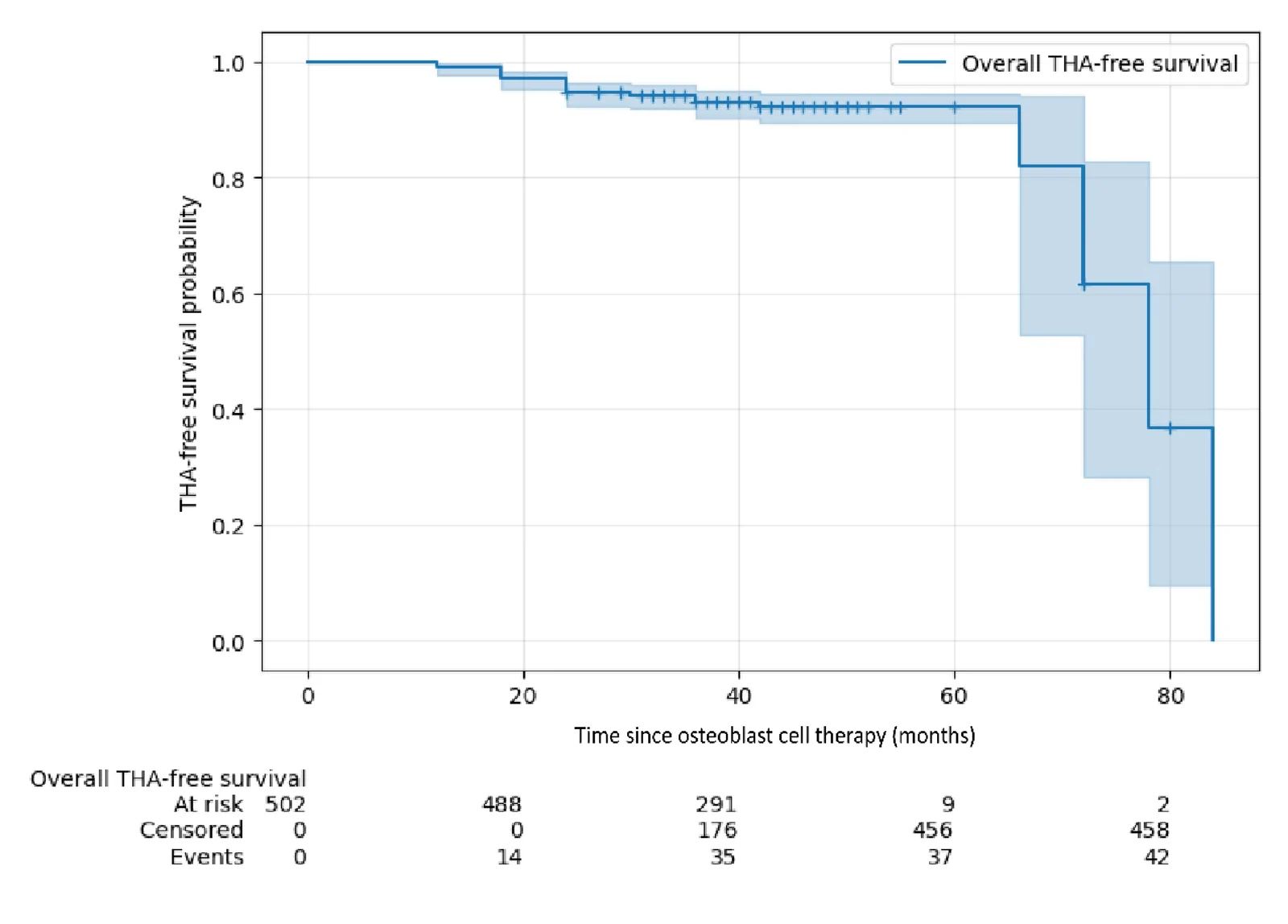
Kaplan–Meier curve for overall THA-free survival for hips THA: total hip arthroplasty.

Based on the survival analysis, the median THA-free survival was 78 months (Figure 9), whereas the median follow-up by the reverse KM method was 43 months (IQR: 39-46 months; range: 12-84 months).

**Figure 10:**
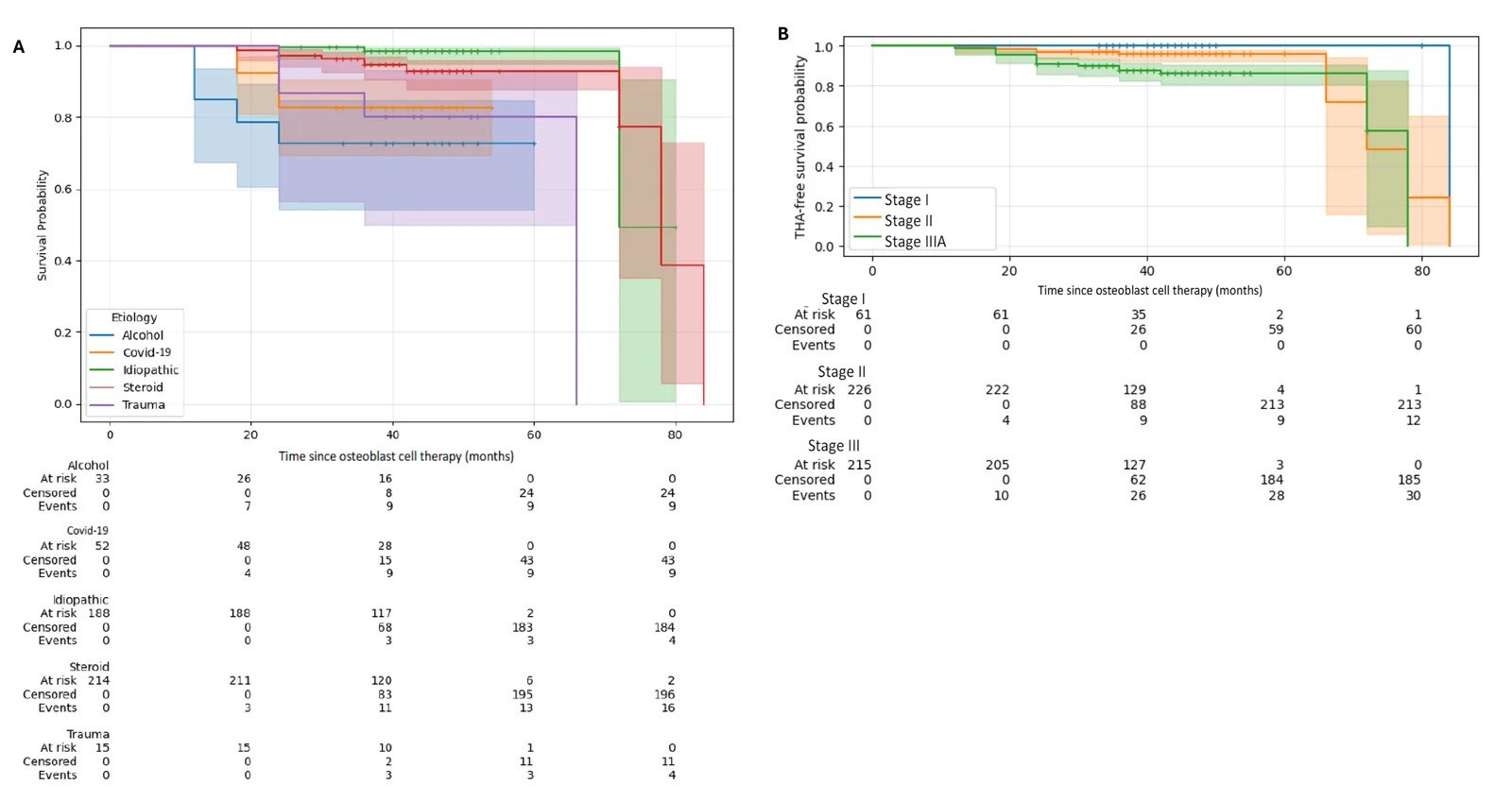
Kaplan–Meier curve for THA conversion at hip level based on (A) etiology and (B) disease stage COVID-19: coronavirus disease 2019; THA: total hip arthroplasty

Furthermore, the etiology-based KM curve showed that trauma, COVID-19, and alcohol-related ONFH had the highest and earliest THA conversions (global log-rank test p < 0.001), whereas the KM curve based on disease stage showed that patients with stage IIIA ONFH were more likely to require THA (Figure 10).

Risk-factor analysis using Cox regression showed that a longer diagnosis-to-treatment interval was a significant risk factor for conversion to THA with a HR of 1.28 per month (95% CI: 1.15-1.41, p < 0.005) (Table 3).

**Table 3:** Risk factor analysis for THA conversion using Cox regression Data are presented hip-wise; p < 0.05 is considered as significant. Bilateral, alcohol, and stage I are considered as reference for laterality, etiology, and stage. CI: confidence interval; COVID-19, coronavirus disease 2019; HHS, Harris Hip Score; HR, hazards ratio; ONFH: osteonecrosis of the femoral head; THA, total hip arthroplasty; VAS, visual analogue score

Parameters	HR (95% CI)	p-value
Duration between diagnosis and treatment (months)	1.28 (1.15-1.41)	< 0.005
Age (years)	1.04 (0.99-1.09)	0.09
Sex	0.53 (0.24-1.19)	0.13
Stage II	4.27 (0.51-35.49)	0.18
Stage IIIA	13.33 (1.56-113.76)	0.02
COVID-19	0.43 (0.15-1.21)	0.11
Steroid	0.19 (0.07-0.49)	< 0.005
Idiopathic	0.09 (0.03-0.30)	< 0.005
Trauma	0.66 (0.17-2.57)	0.55
Unilateral ONFH	0.22 (0.08-0.67)	0.01
Baseline HHS	1.02 (0.98-1.06)	0.43
Baseline VAS	1.08 (0.78-1.51)	0.63

The proportional hazards assumption was assessed using Schoenfeld residuals and global tests, which showed sex and baseline HHS violations. Further, when adjusted for demographic, clinical, and disease factors, regression analysis showed an increased risk of THA conversion with each one-month delay in treatment (odds ratio (OR): 1.39; 95% CI: 1.22-1.61). Delay was examined using clinically meaningful increments (three- and six-month effects) and found to be exponential, for a six-month delay (OR: 7.13; 95% CI: 3.29-15.41) and for a 12-month delay (OR: 50.78; 95% CI: 10.85-237.59). Sensitivity analysis using restricted cubic splines in Cox models confirmed a statistically significant nonlinear association between treatment delay and THA conversion (likelihood ratio test p = 0.0006).

The spline findings indicated disproportionately steeper risk increases at longer delays, with wider uncertainty at the extremes due to fewer observations. Further analysis stratified by disease stage and etiology was conducted using adjusted Cox regression for the full set of covariates. In these analyses, stage IIIA disease remained strongly associated (adjusted HR: 13.60; 95% CI: 2.41-76.72, p = 0.003) with conversion to THA per six-month delay in treatment compared with stage I, while steroid-associated (adjusted HR: 0.22; 95% CI: 0.06-0.84, p = 0.026), and idiopathic (adjusted HR: 0.09; 95% CI: 0.02-0.36, p < 0.001) osteonecrosis were associated with lower hazards of THA compared with alcohol-associated osteonecrosis (Table 4). Overall, these adjusted findings confirm that alcohol is associated with the highest risk etiology for THA conversion in this cohort.

**Table 4:** Adjusted Cox regression hazards modeling per six-month delay in treatment Data are presented hip-wise; p < 0.05 is considered as significant. ^a^Analysis for ARCO stage was adjusted for treatment delay (three- and six-month increments), age, sex, etiology, bilaterality, baseline HHS, and baseline VAS, whereas, for etiology-stratified Cox regression, analysis was adjusted for ARCO stage, treatment delay (three- and six-month increments), age, sex, bilaterality, baseline HHS, and baseline VAS. ARCO: Association Research Circulation Osseous; CI: confidence interval; COVID-19: coronavirus disease 2019; HR: hazards ratio; NA: Not applicable; THA: total hip arthroplasty

Parameters	Number of Hips	THA events	Unadjusted HR (95% CI)	p-value	Adjusted HR (95% CI)^a^	p-value
ARCO 2019 Stage						
Stage I	61	1	1	NA	1	NA
Stage II	226	13	5.82 (0.95-35.68)	0.057	4.30 (0.67-27.68)	0.125
Stage IIIA	215	30	17.55 (3.59-85.78)	< 0.001	13.60 (2.41-76.72)	0.003
Etiology						
Alcohol	33	9	1	NA	1	NA
Steroid	214	18	0.17 (0.05-0.53)	0.002	0.22 (0.06-0.84)	0.026
COVID-19	52	9	0.56 (0.16-1.93)	0.36	0.51 (0.11-2.38)	0.39
Idiopathic	188	4	0.06 (0.02-0.20)	< 0.001	0.09 (0.02-0.36)	< 0.001
Trauma	15	4	0.73 (0.17-3.17)	0.67	0.79 (0.21-3.05)	0.74

Cox proportional hazards model demonstrated that each additional month of delay in osteoblast cell therapy increased the risk of THA (HR: 1.28; 95% CI: 1.15-1.41). A similar estimate was observed after accounting for within-patient correlation and center effect (HR: 1.26; 95% CI: 1.13-1.39). Details of patients who underwent THA for treatment failure are presented in Table 5.

**Table 5:** Details of hips that underwent THA after osteoblast cell therapy ^a^Hip stages were classified per the ARCO 2019 classification. ARCO 2019: 2019 revised version of the Association Research Circulation Osseous; COVID-19: coronavirus disease 2019; F: female; M: male; ONFH: osteonecrosis of the femoral head; THA: total hip arthroplasty.

Sr. No.	Age/sex	Hip involvement (ONFH) diagnosed at the time of osteoblast cell therapy	Stage of disease at the time of osteoblast cell therapy^a ^(Left)	Stage of disease at the time of osteoblast cell therapy^a ^(Right)	Etiology of ONFH	Hip converted to THA (treatment failure)	Time to THA conversion (years)
1	29/F	Bilateral	IIIA	IIIA	Steroid	Right	6
2	38/M	Bilateral	II	IIIA	Steroid	Right	2.5
3	42/M	Bilateral	IIIA	II	COVID-19	Left	1.5
4	36/F	Bilateral	IIIA	II	Steroid	Left	2
5	36/M	Unilateral	-	II	Idiopathic	Right	2
6	29/M	Bilateral	II	IIIA	Alcohol	Left & Right	1
7	34/F	Unilateral	II	-	Trauma	Left	3
8	31/M	Bilateral	II	IIIA	Steroid	Right	1.5
9	36/M	Unilateral	-	II	Idiopathic	Right	3
10	41/M	Bilateral	II	IIIA	Steroid	Right	2
11	36/F	Bilateral	II	IIIA	Steroid	Right	2.5
12	37/M	Bilateral	IIIA	IIIA	Steroid	Left & Right	1.5
13	51/M	Bilateral	II	IIIA	Trauma	Left & Right	2
14	47/M	Bilateral	IIIA	IIIA	COVID-19	Left & Right	2
15	40/M	Bilateral	IIIA	II	Idiopathic	Left	3
16	21/F	Bilateral	IIIA	IIIA	Steroid	Left & Right	3
17	27/M	Bilateral	IIIA	II	Alcohol	Left & Right	1
18	26/F	Bilateral	II	IIIA	Steroid	Right	2
19	39/M	Bilateral	II	IIIA	Alcohol	Left & Right	1.5
20	37/M	Bilateral	IIIA	II	Steroid	Left	3
21	38/F	Bilateral	II	IIIA	COVID-19	Right	1.5
22	45/M	Bilateral	IIIA	II	COVID-19	Left	2
23	46/M	Bilateral	II	IIIA	Alcohol	Left & Right	2
24	29/M	Bilateral	II	I	Idiopathic	Left	6
25	38/F	Bilateral	IIIA	IIIA	COVID-19	Left & Right	2
26	27/M	Bilateral	I	II	Steroid	Left & Right	7
27	24/M	Bilateral	II	IIIA	Steroid	Left & Right	6.5
28	19/F	Bilateral	IIIA	IIIA	Steroid	Left & Right	3.5
29	44/F	Unilateral	-	II	Trauma	Right	5.5
30	37/M	Bilateral	II	IIIA	COVID-19	Left & Right	1.5
31	31/M	Bilateral	IIIA	II	Alcohol	Left	1

## Discussion

We evaluated the clinical outcomes of patients treated with osteoblast cell therapy following core decompression for ONFH (ARCO 2019) in multiple centers across India. The results of the study suggested meaningful improvements in pain intensity and functional capacity, as suggested by the significant mean change in VAS and HHS scores, which exceeded the presupposed MCID at a mean follow-up of 3.5 years. Furthermore, less than 10% of patients (or hips) underwent THA at follow-up, indicating that in young patients, osteoblast cell therapy was associated with slower disease progression, reduced pain, a clinically meaningful improvement in HHS, and delayed need for THA. The median THA-free survival was 78 months despite a small number of late events, and sensitivity analyses supported the robustness of the estimate.

Core decompression is the preferred approach, performed via drilling and eliminating the necrotic lesion. A retrospective single-center study examined the effectiveness of core decompression in 46 patients (65 hips) with an early stage of ONFH, classified according to the Ficat-Arlet staging system, and reported a significant and long-term palliative effect across all stages; however, prevention of disease progression was observed only in patients with stage I ONFH [22]. Similarly, another retrospective study reported a 58% success rate in 135 patients (207 hips) who underwent core decompression for pre-collapse stage (Ficat stages I and II) ONFH [23]. Furthermore, a meta-analysis of 20 studies and 2,123 hips reported improved clinical outcomes with the addition of stem cell therapy to core decompression in patients with ONFH [24]. Consistent with these findings, combining core decompression with regeneration therapy has been shown to accelerate healing and reduce the risk of femoral head collapse [25]. Consequently, core decompression with adjuvant implantation of orthobiologics has been introduced to address the cellular pathophysiology of ONFH.

Considering the altered number of MSCs in the hematopoietic tissue and stroma of the bone marrow in patients with osteonecrosis, it was hypothesized that implantation of ex vivo expanded autologous osteoblasts could enhance the tissue repair process, restore lost bone mass, and prevent disease progression [16]. It was found that bone alkaline phosphatase-characterized osteoblasts have greater regenerative potential compared with heterogeneous bone marrow cells [26,27]. Thus, osteoblast cell therapy could offer the advantages of biological augmentation of core decompression with the implantation of a precise number of osteoblasts (48-50 million cells per joint), which would further enhance the repair and regeneration process. Other advantages of this approach are as follows: (i) the osteoblasts are derived from the patient’s own harvested bone marrow, making the therapy safe for implantation; (ii) removal of necrotic bone creates more space for the formation of new bone; and (iii) the repair and regeneration process is further facilitated because the injected osteoblasts readily integrate with the immature bone tissue from adjacent tissue, while the organized hematoma developed at the decompression site provides a scaffold for the autologous cultured osteoblasts. Several studies have demonstrated the efficacy of implantation of ex vivo expanded autologous osteoblasts with core decompression across various stages of the disease and etiologies [17,28-35]

The results of the present study also indicated an improvement in functional outcomes with the combination of core decompression and osteoblast cell therapy, which is in accordance with the findings from previous research [16,17]. Results from a retrospective observational study showed an improvement in VAS scores from 58.8 ± 13.8 to 32.2 ± 32.1 and HHS scores from 47.1 ± 12.3 to 63.7 ± 27.7 in 61 patients (98 hips) at a mean follow-up of 6.3 years [16]. Recently, Patro et al. evaluated the efficacy of osteoblast cell therapy following core decompression for the treatment of ONFH in 26 patients at a 36-month follow-up [17]. Significant improvements in VAS scores (9.00 ± 0.00 vs 3.01 ± 0.03; p < 0.001) and HHS scores (46.12 ± 3.68 vs 89.23 ± 2.19; p < 0.001) were reported. MRI assessment demonstrated significant osteogenesis at the site of osteoblast cell therapy in 22 (out of 26) patients using MRI. The researcher also reported that the use of osteoblast cell therapy for ONFH treatment represents an innovative regenerative medicine approach aimed at promoting osteogenesis through differentiated osteoblast cells. A key strength of this method is the precise regulation of cell quantity and quality, which enhances the consistency and reliability of therapeutic outcomes [17]. In the present study, we also noted imaging findings suggestive of osteogenesis in the femoral head, with preservation of joint space sphericity, on postoperative MRI images of patients at 24-month and 36-month follow-ups. Furthermore, postoperative X‑ray images of different patients showed radiographic features consistent with osteogenesis at different time intervals. Hence, the present study supports the findings of the previous study that adjuvant osteoblast cell therapy with core decompression can lead to successful bone remodeling and integration of the implant with the host bone tissue [17].

The natural progression of the disease requires THA in up to 75% of patients, as reported in a retrospective study after a 10-year follow-up of patients with ONFH [36]. However, osteoblast cell therapy appears effective in reducing the need for THA [10]. In the present study, after a mean follow-up of 3.5 years, THA conversion was required for 8.8% of treated hips (9.7% of patients); the majority of hips that underwent THA were in stage III (13.95%) and had trauma, COVID-19, or alcohol-related etiologies, which is lower than previously reported [16]. Interestingly, a delay in treatment was a significant finding in our study, with each additional month of delay increasing the hazard of THA by 1.28. In comparison, a previous study reported THA conversion in 28.7% of hips at a mean follow-up of 6.3 years [16]. Also, the patient-to-patient heterogeneity in isolated MSCs could be due to age factor; however, this variability was partially mitigated in the present study through ex vivo expansion and osteogenic differentiation under standardized cell culture conditions, followed by a predefined minimal dose of 48 million viable osteoblast lineage cells transplanted. Therefore, no apparent age-related correlation with study outcomes was observed in the present study.

The major limitations of this study include its retrospective design and lack of a control group. The retrospective design increases the risk of selection bias and limits the ability to adjust for unmeasured confounders and may have contributed to the low reported rate of THA requirement. Moreover, the absence of a control or comparator group does not allow for drawing causal inferences regarding treatment outcomes. In addition, because osteoblast implantation was performed concomitantly with core decompression, the study design did not allow isolation of the independent therapeutic effect of osteoblast cell therapy or evaluation of its incremental benefit over core decompression alone. These limitations should be considered when interpreting the findings. Non-treatment factors such as lifestyle changes, rehabilitation, concurrent medications, or supportive care could have contributed to improvement, but these were not fully accounted for. The surgical procedure and radiological assessments were standardized; however, considering the multicenter nature of the study, methodological, radiological, and rehabilitative heterogeneity should also be considered, which may affect the uniformity of the outcomes. Quantitatively demonstrating application and adherence differences by center, along with incorporating the center effect into the model, would have enhanced the generalizability of the findings.

Given the inclusion of patients with ONFH from various etiological factors, there may be some variability in baseline patient characteristics. Particularly, patients with COVID-19 differed in infection severity and treatment exposure. VAS and HHS were recorded at the patient level and could not be uniquely attributed to individual hips in bilateral disease. Therefore, they were not directly modeled towards hip-level covariates. Since radiological assessment was not performed for all patients and quantitative analysis was unavailable, comprehensive evaluation of radiological disease progression and treatment response was limited. Our analysis suggests exponential effects for delay-to-treatment analysis, which are highly susceptible to confounding by indication and baseline severity; patients treated later may systematically differ in stage distribution, lesion characteristics, etiology, socioeconomic access, and preoperative function. Despite these limitations, the study provides supportive evidence of the efficacy and safety of osteoblast cell therapy for the treatment of ONFH.

## Conclusions

Clinical evidence from a large population data shows that core decompression with adjuvant osteoblast cell therapy was associated with preservation of the femoral head integrity. However, future research, including well‑designed controlled studies with extended follow-up and standardized imaging protocols, with stratification of patients based on etiology and disease grade, is recommended.
